# Interplay of recombination and selection in the genomes of *Chlamydia trachomatis*

**DOI:** 10.1186/1745-6150-6-28

**Published:** 2011-05-26

**Authors:** Sandeep J Joseph, Xavier Didelot, Khanjan Gandhi, Deborah Dean, Timothy D Read

**Affiliations:** 1 Department of Medicine, Division of Infectious, Diseases Emory University School of Medicine, 615 Michael Street, Atlanta, GA 30322, USA; 2 Department of Human Genetics, Emory University School of Medicine, 615 Michael Street, Atlanta, GA 30322, USA; 3 Department of Statistics, University of Oxford, Oxford, OX1 3TG, UK; 4 Center for Immunobiology and Vaccine Development, Children's Hospital Oakland Research Institute, Oakland, California, USA; 5 Department of Medicine, University of California, San Francisco, San Francisco, California, USA; 6 Joint Graduate Program in Bioengineering, University of California, San Francisco, California, USA; 7 University of California, Berkeley, Berkeley, California, USA

## Abstract

**Background:**

*Chlamydia trachomatis *is an obligate intracellular bacterial parasite, which causes several severe and debilitating diseases in humans. This study uses comparative genomic analyses of 12 complete published *C. trachomatis *genomes to assess the contribution of recombination and selection in this pathogen and to understand the major evolutionary forces acting on the genome of this bacterium.

**Results:**

The conserved core genes of *C. trachomatis *are a large proportion of the pan-genome: we identified 836 core genes in *C. trachomatis *out of a range of 874-927 total genes in each genome. The ratio of recombination events compared to mutation (ρ/θ) was 0.07 based on ancestral reconstructions using the ClonalFrame tool, but recombination had a significant effect on genetic diversification (r/m = 0.71). The distance-dependent decay of linkage disequilibrium also indicated that *C. trachomatis *populations behaved intermediately between sexual and clonal extremes. Fifty-five genes were identified as having a history of recombination and 92 were under positive selection based on statistical tests. Twenty-three genes showed evidence of being under both positive selection and recombination, which included genes with a known role in virulence and pathogencity (e.g., *ompA, pmps, tarp*). Analysis of inter-clade recombination flux indicated non-uniform currents of recombination between clades, which suggests the possibility of spatial population structure in *C. trachomatis *infections.

**Conclusions:**

*C. trachomatis *is the archetype of a bacterial species where recombination is relatively frequent yet gene gains by horizontal gene transfer (HGT) and losses (by deletion) are rare. Gene conversion occurs at sites across the whole *C. trachomatis *genome but may be more often fixed in genes that are under diversifying selection. Furthermore, genome sequencing will reveal patterns of serotype specific gene exchange and selection that will generate important research questions for understanding *C. trachomatis *pathogenesis.

**Reviewers:**

This article was reviewed by Dr. Jeremy Selengut, Dr. Lee S. Katz (nominated by Dr. I. King Jordan) and Dr. Arcady Mushegian.

## Background

Genetic variation is essential for the long term survival of bacteria [1]. Mutation and recombination are the basic processes by which genetic variation emerges in bacterial populations [2]. Recombination is the incorporation into a genome of imported DNA, and leads to the introduction of novel sequences in the chromosome as well as the creation of loci with mosaic genes (new haplotypes) [2-4]. Recombination plays a major role in the potential for adaptation of a bacterial population [1,5,6]. The traces of homologous recombination have been detected through comparative analysis of the genomes of many bacterial pathogens including *Streptococcus spp *[1], *Listeria monocytogenes *[7]*Campylobacter *[8], *Escherichia coli *[9], *Chlamydia spp *[10] and *Salmonella spp *[11], and can constitute a much faster mode of evolution than point mutations [4,12,13]. On the other hand, analysis of selective pressures based on the relative rates of non-synonymous and synonymous mutations of natural bacterial populations has revealed the direction and strength of natural selection on coding sequences [8,14]. Several gene-specific [15-22] as well as whole genome analyses [1,7,11,23-26] of pathogenic bacterial populations have suggested positive selection in virulence genes that enhance host infection potential.

At the population level, natural selection acts on the basic processes of mutation and recombination, causing some events to be quickly purged from the population and others to become widespread [2]. In a perfectly clonal population, every advantageous mutation is linked to other alleles in the genome [7,14]. Recombination can play an important role in evolution by combining advantageous mutations and thereby assisting in their fixation [7,14]. During the adaptation of a bacterial population to a new ecological niche, selective pressures are shifted, resulting in a higher or lower rate of recombination and/or changes in genetic flux, depending on the exact conditions [2]. Recombination and positive selection are therefore two important evolutionary forces in microbial pathogens that drive adaptation to new hosts, resistance to the action of antibiotics and promote survival in the face of host immune challenge [1,7,11,16,27].

*Chlamydia trachomatis *is an obligate intracellular bacterial species that causes ocular diseases and sexually transmitted diseases in humans worldwide [28]. However, progress toward understanding the biology of *C. trachomatis *has been hindered by the requirement for intracellular growth and the lack of a routine system for directed mutagenesis [10]. *C. trachomatis *isolates are differentiated into serovars based on serospecificity for the major outer membrane protein (MOMP; encoded by the gene *ompA*) [29]. Serovars that are restricted to the epithelial cells of the eye and genital tract include: (1) ocular, trachoma serovars A to C and Ba; although B, Ba and C serovars are found in the genital tract but less frequently; (2) non-invasive sexually transmitted disease causing serovars D to K, Da, Ia, and Ja; these serovars also infect the conjunctiva and cause a self-limited conjunctivitis; and (3) invasive serovars L_1_, L_2_, L_3 _and L_2_a and L_2_b that cause lymphogranuloma venereum (LGV). L_2_b has been described recently in Europe and North America as an outbreak strain responsible for hemorrhagic proctitis [30]. To date, there is only one whole-genome based analysis (limited to three *C. trachomatis *strains) that investigated selective pressures by calculating the ratio of the number of synonymous and non-synonymous mutations [26], while gene-based experiments identified the gene coding for major outer membrane protein (*ompA*) to be under natural selection [31-35]. Evidence for recombination and recombination hotspots were initially identified in gene-based studies [36-39]. Experimental studies using a naturally occurring ofloxacin resistant strain (with mutation in *gyrA*) and a naturally occurring rifampacin resistant strain (with mutation in *rpoB*) resulted in the isolation of a strain resistant to both antibiotics that carried both mutations [39,40]. Similarly it was shown that nonfusogenic strains of *C. trachomatis *that lack inclusion membrane protein A (IncA), a protein involved in homotypic inclusion fusion, still recombine *in vitro *when subjected to the same selection parameters [41]. Previous studies based on whole-genome analysis used limited strains [10] and MLST analysis [42] to identify potential recombination targets in *C. trachomatis*. We recently identified a recombinant clinical isolate isolated from a man who has sex with men (MSM) comprised of an invasive LGV strain and a non-invasive common urogenital strain D that, on genome-wide analysis, was found to have seven discrete regions of genetic exchange, including genes involved in inclusion formation and replication [43]. However, the magnitude of recombination in *C. trachomatis *on a genome-wide level using multiple strains has not been characterized before.

Advances in genome sequencing technologies [44] have facilitated the generation of whole-genome sequences of the various *C. trachomatis *serovars, which paved the way to look into the population biology of this commonly encountered yet still mysterious bacterium [10,28]. Understanding the evolutionary processes behind disease tropism and severity in *C. trachomatis *strains will lead to better use of medical countermeasures. One question of particular importance is whether mutations arising from selection in genes for host tropism or increased virulence may be transmitted frequently to other members of the species through recombination. In order to assess the contributions of recombination and selection in this pathogen, we performed a comparative analysis of 12 published complete *C. trachomatis *genomes.

## Materials and methods

### Genome data and Ortholog retrieval

Our data consisted of the complete sequences of twelve finished genomes of *Chlamydia trachomatis *and one genome of *Chlamydia muridarum*, obtained from GenBank (Table 1). Coding sequences were extracted from NCBI RefSeq annotation and the orthologs were determined using OrthoMCL [45]. OrthoMCL uses reciprocal best BLASTp scores in a normalized similarity matrix that is analyzed using an additional step of Markov Clustering to improve sensitivity and specificity. OrthoMCL was run with a BLAST E-value cut-off of 1e-05, and an inflation parameter of 1.5. We defined the core genes as the orthologous genes that are shared by all *C. trachomatis *strains.

**Table 1:** List of *C. trachomatis *used in this study.

Abbreviation	*C. trachomatis *Strains Used	GenBank Accession	Number of proteins	Genome Size (bp)
*L*_*2*_	*C. trachomatis 434/Bu*	AM884176	874	1038842

*L*_*2*_*b*	*C. trachomatis L*_*2*_*b/UCH-1/proctitis*	AM884177	874	1038869

*A/Har13*	*C. trachomatis A/HAR-13*	CP000051	911	1044459

*B/Jali*	*C. trachomatis B/Jali20/OT*	FM872308	875	1044352

*B/Tz*	*C. trachomatis B/TZ1A828/OT*	FM872307	880	1044282

*D/UW3*	*C. trachomatis D/UW-3/CX*	AE001273	895	1042519

*E/11023*	*C. trachomatis E/11023*	CP001890	926	1043025

*E/150*	*C. trachomatis *E/150	CP001886	927	1042996

*G/11222*	*C. trachomatis G/11222*	CP001888	927	1042354

*G/11074*	*C. trachomatis G/11074*	CP001889	919	1042875

*G/9301*	*C. trachomatis G/9301*	CP001930	921	1042811

*G/9768*	*C. trachomatis G/9768*	CP001887	920	1042810

*Ng*	*Chlamydia muridarum *(out group)	AE002160	904	1072950

### Alignments and Phylogenetic Inference

**Figure 1:**
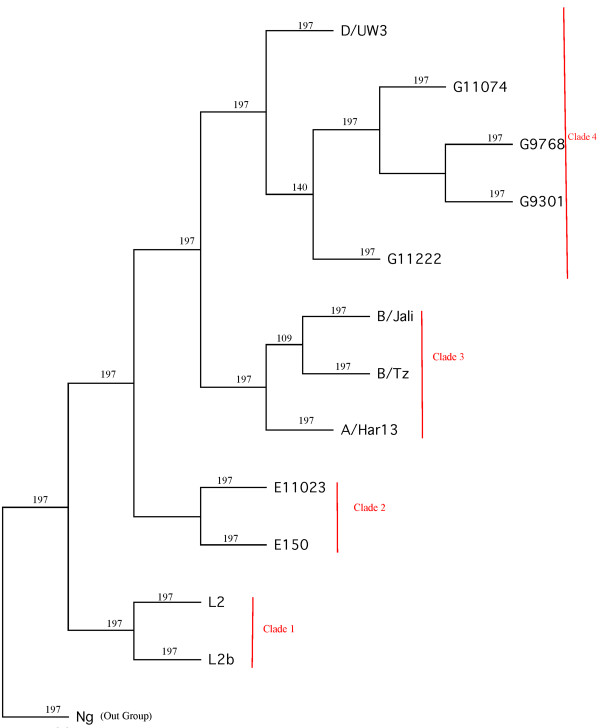
**Phylogeny of *Chlamydia trachomatis***. Twelve *C. trachomatis *strains along with one *C. muridarum *(out-group) are represented in this tree. The phylogeny was constructed based on the concatenated alignment of all individual core genes (286,496 amino acid sites) by both NJ and MP procedures. Both NJ and MP analysis inferred the same tree. The numbers represent bootstrap values.

The program MUSCLE [46] was used for multiple sequence alignment (MSA) of the core protein-coding genes using default settings. The resulting protein alignments were reverse-translated to codon-based nucleotide alignments using PAL2NAL [47], which used the corresponding DNA sequences for positive selection analysis (see below), and another set of protein alignments were filtered by GBLOCKS [48] using default settings to remove regions that contained gaps or were highly divergent. Phylogenetic analyses were conducted using the TREE-PUZZLE [49] and PHYLIP [50] packages. The evolutionary distances were computed by TREE-PUZZLE where the parameters were set to the JTT substitution model, the mixed model of rate heterogeneity with one invariant and eight gamma rate categories, and using the exact and slow parameter estimation. The level of bootstrap support for each gene was inferred by 200 resamplings of the alignment using SEQBOOT in the PHYLIP package. Whole-genome trees (species tree) were constructed based on the concatenated alignment of all individual core genes following the Neighbor-Joining (NJ) and maximum parsimony (MP) methods. NJ trees were constructed using NEIGBOR in the PHYLIP package and the extended majority rule consensus tree was inferred for each core gene. The MP tree was constructed using PROTPARS in the PHYLIP package with 100 randomizations of input order and 4 clades were defined (Figure 1).

### Analysis of Positive Selection

Genes under positive selection were identified using codeml as implemented in PAML version 4.4 [51]. Two types of tests were implemented in PAML to identify genes under positive selection: Test 1 was carried out using the null model M1a (Nearly-neutral) and the alternative model M2a (positive selection) [52], whereas Test 2 was carried out to identify genes under positive selection in specific branches of *C. trachomatis *(branch-site test2 described by Zhang et al. [53]). Test 1 identified genes under positive selection in any or all of the branches of a given phylogeny while Test 2 identified genes under positive selection in the four clades of the whole-genome (species) tree (Figure 1). Initially, the inferred whole-genome tree was used for all PAML analyses. For all genes that were identified as being under positive selection, Test 1 and Test 2 PAML were re-run to check if the positive selection results obtained using gene-specific trees differed from that of the whole genome tree (Figure 1). For each test, the likelihood of a model that does not allow positive selection (null model) was compared to a model that allows positive selection (alternative model) using a Likelihood Ratio Test (LRT) [53]. For branch-specific tests (Test 2), one degree of freedom was used to calculate p-values, while for the overall test (Test 1), 2 degrees of freedom were used to calculate p-values. Correction for multiple testing was performed using the Benjamini & Hochberg method [54] implemented in the software Q-value [55].

### Analysis of intragenic recombination

To detect homologous intragenic recombination in the gene clusters, we implemented the following four methods: **(1) **Pairwise Homoplasy Index (PHI) **(2) **Neighbor Similarity Score (NSS) **(3) **Maximum χ2, and **(4) **Sawyer's Runs test [56,57]. The first three methods were implemented using the PhiPack package [6] while the Sawyer's Runs test was implemented in GENECONV version 1.81a. For PHI, a window size of 50 nucleotides was used. For Maximum χ2, a fixed window size of two thirds of the number of polymorphic sites was used. For GENECONV analyses, the parameter g-scale was set to 1 and only the inner fragments were considered. P-values were estimated by employing 10,000 permutations of the alignment for GENECONV and 1,000 permutations for the other three methods. For the above four recombination detection methods, we removed *C. muridarum *genes because of the much greater evolutionary separation of this genome from the other twelve. MSAs on the clusters identified by OrthoMCL minus the *C. muridarum *genes were recomputed using MUSCLE.

### Evolutionary analysis using ClonalFrame

ClonalFrame [58] is a statistical approach to reconstructing the clonal genealogy while identifying genomic fragments that have been affected by recombination and accounting for their disrupting effect. A whole genome sequence alignment of the 12 *C. trachomatis *genomes was created using progressive Mauve [59,60] and the core alignment was extracted by keeping only the regions where all genomes aligned over at least 500 bp. Four independent runs of ClonalFrame were performed, each consisting of 40,000 iterations. The first half of these iterations was discarded as MCMC burn-in. Good convergence and mixing properties were found by manual comparison between the four runs, making sure that they produced consistent estimates of the global parameters, clonal genealogy and location of recombination events.

We defined putative imports for each branch of the ClonalFrame tree as a genomic region for which the probability of recombination never goes below 80% and reaches 95% in at least one site. Such putative imports were extracted from each of the 4 clades, and for each of them we searched for the strain(s) with the highest normalized BLASTN score along with a percent identity (pcident) of at least 98% in the whole database containing all the 'finished' genome and plasmid sequences of *Chlamydiaceae *bacterial species minus the strains of the clade affected by the import. If all the best hits were with strains belonging to the same clade, the origin of the event was attributed to this clade, and otherwise the origin was called ambiguous.

### Calculation of Linkage Disequilibrium (LD)

To illustrate the distance-dependent decay of LD, we adopted the method implemented by Sharpiro et al., 2009 [14]. We used the core orthologs (836 genes) that are present in one copy per genome in each of the 12 *C. trachomatis *strains and each unique allele was assigned a unique allele number. We then selected pairs of loci separated by increasing distance in the *C. trachomatis *D/UW-3/CX reference genome. Neighboring loci on the same strand were excluded. LD between 2 pairs of loci was estimated using the *D*'_*A *_metric [61]

which provides a summary measure of LD between two loci, with allele frequencies *p *and *q*, each containing *m *and *n *number of arbitrary alleles and *D*'_*ij *_represents the normalized amount of linkage disequilibrium that accounts for the variation of the allele frequencies. When *D*'_*A *_= 1, linkage is at its theoretical maximum.

## Results

### Ortholog Identification and Genome Phylogeny of *C. trachomatis*

In order to improve our understanding of the evolution and population structure of *C. trachomatis*, we performed full genome analyses for homologous recombination and positive selection using the complete and published genomes of twelve *C. trachomatis *strains. Our analysis for recombination and positive selection was focused on the core genome. From the 12 *C. trachomatis *strains and the one *C. muridarum *(strain Ng, out-group) (Table 1) used in this study, we identified 818 single-copy genes shared by all 13 strains. After excluding *C. muridarum*, the number of single-copy genes went up to 836 genes shared by all 12 *C. trachomatis *strains. Core genes represent more than 90% of the annotated genes from the smallest genome (L_2 _and L_2_b strains) used in this study (Table 1). This result is in line with the known high level of synteny and genome sequence conservation [28]. The analysis also shows that the mouse-infecting species *C. muridarum *shares more than 90% of the core gene set of *C. trachomatis*. It has been suggested that the majority of apparent disparity in gene content might be caused by differences in the gene predictions rather than being real differences in the presence or absence of genes [28]. For the purpose of phylogenetic inference, we used the 818 single-copy genes shared by all 13 strains.

**Figure 2:**
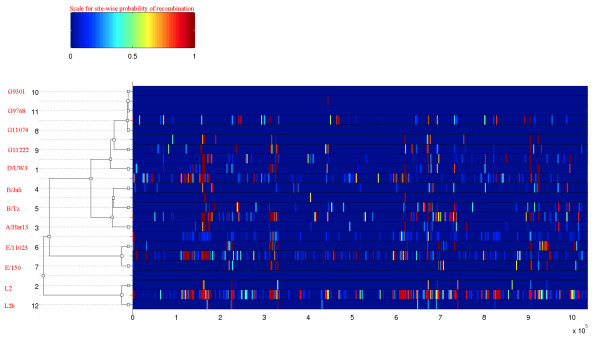
**Results of the ClonalFrame analysis on an alignment of the 12 *C. trachomatis *genomes**. The inferred clonal genealogy is shown on the left. To each branch of this tree corresponds a row of the heat map, which is vertically aligned with it. Each row of the heat map shows the posterior probability of recombination estimated by ClonalFrame on the corresponding branch (Y-axis) and along the positions of the alignment (X-axis). These probabilities are color-coded according to the legend shown at the top.

The phylogeny of *C. trachomatis *was inferred using two different approaches. The first approach utilized a concatenated alignment of 286,496 amino acid residues of the 818 translated core genes followed by NJ and MP phylogenetic inference, and the second approach was based on ClonalFrame analysis. Both approaches yielded trees with the same topology (Figures 1 &2), based on which we defined the following four clades (Figure 1): clade 1 contained L_2 _and L_2_b (LGV strains), clade 2 contained E/11023 and E/150 (non-invasive prevalent sexually transmitted strains), clade 3 contained B/Tz, B/Jali and A/HAR13 (trachoma strains) and clade 4 contained D/UW3, G11074, G11222, G/9301 and G9768. Clade 4 contained both non-invasive prevalent (D/UW3) and non-prevalent (G) strains.

### Substitution analysis reveals widespread recombination across the *C. trachomatis *genome

**Figure 3:**
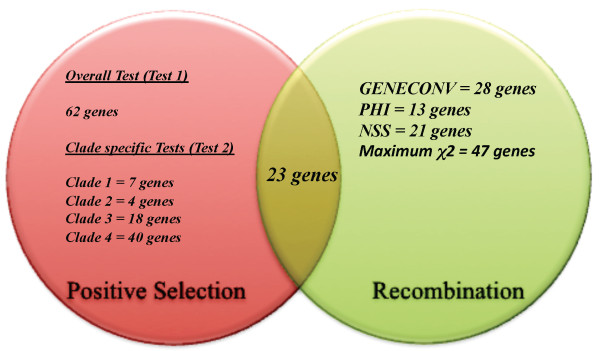
**Venn diagram showing the number of genes under positive selection and recombination**. These genes were identified to be under positive selection based on test 1 (overall test) and test 2 (clade-specific test) along with the number of genes identified as undergone recombination by each of the four-recombination methods implemented. Twenty-three genes were identified to be both under recombination and positive selection.

Based on the four-substitution analysis methods (PHI, NSS, Maximum χ2 and GENECONV) implemented for detecting intragenic homologous recombination, 55 genes showed significant evidence for recombination (p < 0.05) in at least one of the four-recombination analysis performed. GENECONV, PHI, NSS and Maximum χ2 respectively identified 28, 13, 21 and 47 orthologs with significant evidence for recombination (p < 0.05) (Additional File 1; Figure 3). A total of 24, 16, 5 and 8 orthologs showed significant evidence for recombination in one, two, three and all four methods, respectively (Additional File 1 & Table 2). The 8 genes identified as having undergone recombination by all 4 methods were three polymorphic outer membrane protein genes (*pmpE *(CT869), *pmpF *(CT870) &*pmpH *(CT872)), two hypothetical protein genes (CT049, CT144), Type III secretion structural protein gene (*yscC *(CT674), the major outer membrane protein gene (*ompA *(CT681)) and the gene encoding elongation factor Ts (*tsf *(CT679)) (Table 2). Our analysis also identified *pmpG *(NSS, MaxChi) and *pmpI *(MaxChi) as having undergone recombination. Other membrane proteins detected to have been subject to homologous recombination were *yhgN *family protein gene (CT852), putative integral protein genes (CT580, CT838), inclusion membrane protein D genes (CT115, CT288) and ABC transporter membrane protein gene (CT686). The complete list of genes that showed evidence for gene conversion based on substitution analysis in this study is shown in Table 2 and Additional File 1.

**Table 2:** Gene loci identified as being under intragenic homologous recombination by two or more of the four substitution analysis methods implemented in this study.

Locus Tag	Locus	Gene Annotation	Recombination Method
CT674	*yscC*	Type III secretion structural protein (outer membrane ring)	NSS, MaxChi GeneCon, PHI

CT679	*tsf*	elongation factor Ts	NSS, MaxChi GeneCon, PHI

CT681	*ompA*	major outer membrane protein	NSS, MaxChi GeneCon, PHI

CT869	*pmpE*	polymorphic outer membrane protein	NSS, MaxChi GeneCon, PHI

CT870	*pmpF*	polymorphic outer membrane protein	NSS, MaxChi GeneCon, PHI

CT872	*pmpH*	polymorphic outer membrane protein	NSS, MaxChi GeneCon, PHI

CT049	-	hypothetical protein	NSS, MaxChi GeneCon, PHI

CT144	-	hypothetical protein	NSS, MaxChi GeneCon, PHI

CT675	*karG*	ATP:guanido phosphotransferase	GeneCon, NSS, MaxChi

CT682	*pbpB*	penicillin-binding protein	GeneCon, NSS, MaxChi

CT686	-	hypothetical protein	NSS, MaxChi, PHI

CT456	-	Translocated actin-recruiting phosphoprotein (tarp protein)	NSS, MaxChi, GeneCon

CT619	-	hypothetical protein	NSS, MaxChi, GeneCon

CT640	*recC*	exodeoxyribonuclease V gamma chain	NSS, MaxChi

CT643	*topA*	DNA topoisomerase I/SWI domain fusion protein	MaxChi, GeneCon

### Evidence for Positive Selection

Using PAML we identified 62 (Additional File 2) genes (7.58% of the genome) under positive selection (FDR < 10%) with the overall test (Test 1), while the clade specific tests (Test 2) identified respectively 7, 4, 18 and 40 genes in clades 1, 2, 3 and 4 (Additional File 3; Figure 3). No genes were found to be under positive selection in all 4 clades. A high proportion of positively selected genes in clades 1, 2, 3 and 4 were only under selection in one clade (71%, 77%, 61% and 100% respectively). Test 1 identified 11 genes associated with membrane and transport system associated proteins such as the major outer membrane protein gene (*ompA*), polymorphic outer membrane protein genes (*pmpE *and *pmpF*), putative integral membrane protein gene (CT012, CT147, CT580), inclusion membrane protein genes (CT115, CT288), translocated actin-recruiting protein gene (*tarp*) (CT456) and oligopeptide transport system binding protein genes (*oppA_1 & oppA_3*) to be under positive selection. Nineteen genes were identified as being under positive selection with both Test 1 and Test 2 (Table 3).

**Table 3:** Gene loci that were identified as being under positive selection by both test 1 and test 2.

Locus Tag	Locus	Gene Annotation	Test 1 FDR p-value	Test 2 FDR p-value
CT868	-	hypothetical protein	1.89E-05	0.005 (Clade 1)

CT115	-	inclusion membrane protein D	0.00045	0.0003 (Clade 1)

CT249	-	hypothetical protein	0.0038	0.078 (Clade 1)

CT456	-	hypothetical protein/translocated actin-recruiting protein (tarp)	0.0021	6.3E-06 (Clade 1)

CT011	-	hypothetical protein	0.0088	0.002 (Clade 2)

CT823	*htrA*	serine protease	0.00014	0.007 (Clade 3)

CT012	-	Hypothetical protein/putative integral membrane protein	2.56E-12	2.12E-23 (Clade 3)

CT147	-	Hypothetical protein/putative integral membrane protein	6.31E-14	7.34E-15 (Clade 3)

CT209	*leuS*	leucyl-tRNA synthetase	7.36E-06	0.01 (Clade 3)

CT227	-	hypothetical protein	0.0017	1.63E-07 (Clade 3)

CT244	-	hypothetical protein	2.95E-06	1.56E-08 (Clade 3)

CT604	*groEL_2*	60 kDa chaperonin GroEL2	0.006	1.35E-15 (Clade 4)

CT745	*hemG*	protoporphyrinogen oxidase	0.021	8.32E-07 (Clade 4)

CT840	*mesJ*	tRNA(Ile)-lysidine synthase/PP-loop superfamily ATPase	0.03	0.036 (Clade 4)

CT841	*ftsH*	ATP-dependent zinc protease/Cell division protein	0.06	0.087 (Clade 4)

CT847	-	hypothetical protein	0.031	0.018 (Clade 4)

CT875	-	hypothetical protein	2.93E-07	0.001 (Clade 4)

CT223	-	hypothetical protein	2.95E-06	0.05 (Clade 4)

CT286	*clpC*	ATP-dependent Clp protease	0.019	0.036 (Clade 4)

Among the 92 genes that showed evidence for positive selection by either Test 1 or Test 2, 23 genes (Figure 3, Table 4) also showed evidence for recombination, including 9, 6, 2 and 6 genes showing evidence for intragenic homologous recombination in one, two, three and all four methods, respectively. The correlation between recombination being detected by at least one method (55 genes out of 836) and positive selection being detected by at least one test (92 genes out of 836) was statistically significant (Fisher's Exact Test, p-value < 0.001).

**Table 4:** Gene loci identified to be both under positive selection and homologous recombination.

Locus Tag	*Locus*	Gene Annotation	Positive Selection Analysis	FDR-p-value (Test 2 FDR p-value)	Recombination Method	Smallest significant p-value
CT681	*ompA*	major outer membrane protein	Test 1	0.002606405	NSS, MaxChi GeneCon, PHI	0.00E+00

CT824	-	insulinase family zinc metalloprotease	Test 1	9.81E-05	NSS, PHI	5.00E-03

CT852	*yhgN*	Yhgn Family Protein/putative integral membrane protein	Test 2 (Clade 1)	0.01888237	MaxChi, GeneCon	0.00E+00

CT869	*pmpE*	polymorphic outer membrane protein	Test 1	1.55E-32	NSS, MaxChi GeneCon, PHI	0.00E+00

CT870	*pmpF*	polymorphic outer membrane protein	Test 1	1.55E-32	NSS, MaxChi GeneCon, PHI	0.00E+00

CT875	-	hypothetical protein	Test 1, Test 2 (Clade 4)	2.93E-07 (0.001)	MaxChi	8.00E-03

CT011	-	hypothetical protein	Test 1, Test 2 (Clade 2)	0.008772363	MaxChi, GeneCon	0.00E+00

CT033	*recD_1*	exodeoxyribonuclease V alpha chain	Test 2 (Clade 4)	0.0316283	NSS	4.40E-02

CT049	-	hypothetical protein	Test 1	6.42E-06	NSS, MaxChi GeneCon, PHI	0.00E+00

CT107	*mutY*	A/G-specific adenine DNA glycosylase	Test 1	2.44E-10	MaxChi, GeneCon	3.00E-04

CT112	*pepF*	oligoendopeptidase F	Test 2 (Clade 4)	0.014	GeneCon	0.0127

CT115	*incD*	inclusion membrane protein D	Test 1, Test 2 (Clade 1)	4.51E-04 (3.56E-04)	MaxChi	1.10E-02

CT144	-	hypothetical protein	Test 1	5.78E-13	NSS, MaxChi GeneCon, PHI	0.00E+00

CT244	-	hypothetical protein	Test 1, Test 2 (Clade 3)	2.95E-06 (1.56E-08)	MaxChi, GeneCon	0.00E+00

CT288	-	Hypothetical protein/candidate inclusion membrane protein	Test 1, Test 2 (Clade 4)	2.95E-06 (0.0058)	NSS	5.00E-03

CT315	*rpoB*	DNA-directed RNA polymerase subunit beta	Test 2 (Clade 4)	0.0067	MaxChi	2.10E-02

CT448	*secD/secF*	bifunctional preprotein translocase subunit SecD/SecF	Test 2 (Clade 3)	2.50E-05	MaxChi, GeneCon	0.00E+00

CT456	-	hypothetical protein/translocated actin-recruiting protein (tarp)	Test 1, Test 2 (Clade 1)	0.0021 (6.30E-06)	NSS, MaxChi, GeneCon	0.00E+00

CT470	*recO*	DNA repair protein RecO	Test 1	0.0036	MaxChi	4.00E-03

CT551	*dacC*	D-alanyl-D-alanine carboxypeptidase	Test 2 (Clade 4)	0.051	MaxChi	2.00E-02

CT580	-	hypothetical protein/putative integral membrane protein	Test 1	1.46E-09	MaxChi	1.10E-02

CT604	*groEL_2*	60 kDa chaperonin GroEL2	Test 1, Test 2 (Clade 3)	0.006 (1.35E-15)	MaxChi, GeneCon	0.00E+00

CT619	-	hypothetical protein	Test 2 (Clade 1)	0.008	NSS, MaxChi, GeneCon	0.00E+00

GeneCon -> GENECONV; NSS -> Neighbor Similarity Score; MaxChi -> Maximum χ2; PHI -> Pairwise Homoplasy Test

### Recombination analysis using ClonalFrame

The impact of recombination on *C. trachomatis *genomes was further quantified by applying the ClonalFrame algorithm to whole genome sequences in order to estimate the number of recombination events. ClonalFrame estimated two statistics namely, ρ/θ and r/m, where the former is the ratio of the rates at which recombination and mutation occur while the latter is the ratio of rates at which sites are altered through recombination and mutation. ρ/θ measured how often recombination events happened relative to mutation and r/m measured how important the effect of recombination was in the diversification of the bacterial species relative to mutation [58]. These two statistics can be used to assess the relative contribution of recombination and mutation in the evolution of the strains from a common ancestor.

ClonalFrame estimated the 95% credibility interval of ρ/θ, to be 0.05-0.11 (mean = 0.07). This indicates that mutation, despite an abundance of recombination events, has been the predominant evolutionary process in *C. trachomatis*. The 95% credibility interval of r/m was 0.56-1.01 (mean = 0.71), indicating that, unlike mutation, recombination often affects several nucleotides at each occurrence. The higher rate of r/m illustrates that the impact of recombination on genetic diversity in *C. trachomatis *has been significant. We found the mean tract length of recombined fragments to be 202 bp. This is similar to what has been found in other natural populations of bacteria [2], but is smaller than was observed in laboratory experiments with *C. trachomatis *[37-39]. Such discrepancy in the lengths of recombination events measured *in vivo *and *in vitro *has previously been described in *H. pylori *[62] and *E. coli *[9]. It has been suggested that this difference might be due to the fact that in nature, larger events are more likely to reduce fitness and therefore be purged before observation [2,17]. Figure 2 shows the distribution of recombination events inferred on the branches of the clonal genealogy along the genome of *C. trachomatis*. Ninety-four clear events were found in clade 1, 52 in clade 2, 50 in clade 3 and 62 in clade 4. Clade 1 containing the LGV strains showed the highest number of recombination events that corroborated with previously identified possible inter-strain recombination for LGV [26].

### Inter-clade recombination flux

**Figure 4:**
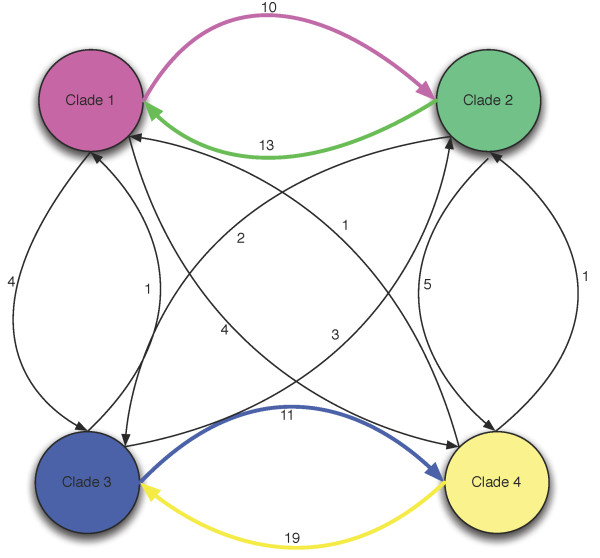
**Inter-clade recombination flux**. Genetic flow between clades. Each arrow is labeled with the number of genetic imports that were inferred from the given origin to the given clade of destination. Clade 1 contained L_2 _and L_2_b (LGV strains), clade 2 contained E/11023 and E/150 (non-invasive prevalent sexually transmitted strains), clade 3 contained B/Tz, B/Jali and A/HAR13 (trachoma strains) and clade 4 contained D/UW3, G11074, G11222, G/9301 and G9768.

ClonalFrame does not model the origin of recombination events [58]. Instead, it requires post-processing of its output to determine where each event is likely to have come from [2,63]. We performed such an analysis as described in the Methods section, and assigned to each recombination event a putative origin. These results provided an insight into the pattern of genetic exchange between *C. trachomatis *clades. Figure 4 summarizes the flow of recombination events with an unambiguous origin that affected members of each clade. Seventy-four inter-clade imports were detected, with more inter-clade DNA exchange between clades 1 and 2 and between clades 3 and 4 indicating uneven historical levels of genetic exchange between clades (Additional File 4).

### Distance-dependent decay of Linkage Disequilibrium (LD) in *C. trachomatis *genomes

**Figure 5:**
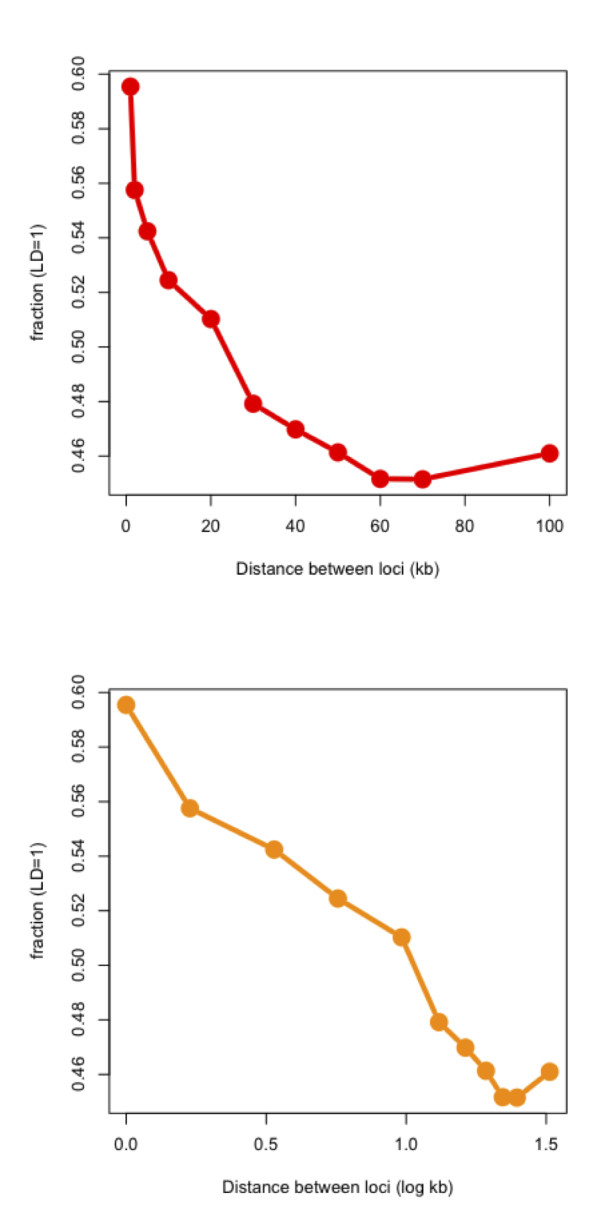
**Illustration of distance-dependent decay in *C. trachomatis *core genome**. **A) **For pairs of loci separated by increasing genetic distance on a linear scale, the proportion of pairs in full linkage (number of pairs with D_A_' = 1 ÷ total number of pairs in that distance bin) is plotted on the y-axis. **B) **The same plot as in figure 5(A) but the distance between loci is shown on a log scale (log kb).

Bacteria show significant diversity in population structure, some being almost completely clonal (e.g. *Staphlococcus aureus*), while others are almost panmictic (e.g. *Neisseria gonorrhoeae*) [14,64]. Previous studies have demonstrated that in many recombining lineages such as *Camplylobacter *[65], *Neisseria *[17], *Helicobacter *and *E. coli*, LD decays with relative distance on the genomes. To illustrate this effect in *C. trachomatis*, we implemented an *in silico *experiment as described in the Methods section. Genes up to ~20 kb apart are found to be linked, but this linkage drops off at approximately 100 kb (Figure 5), similar to what has been observed in a study using the same methodology with 24 *E. coli *strains [14], even though the drop in the actual magnitude of LD is larger in *E. coli *compared to that of *C. trachomatis *(Figure 5).

## Discussion

In this study we used 12 complete published genomes to provide the first global insight into the processes of recombination and positive selection in *C. trachomatis*. The ClonalFrame reconstruction indicated a steady rate of recombination in the history of the organism, estimated at one recombination event for every 5-10 mutations. Moreover, recombination events were distributed throughout the genome. This corroborates with the finding that all contemporary *C. trachomatis *strains have maintained intact *recA *genes and relatively complete recombination systems [66]. It is notable that the summary statistic based detection methods employed here (GENECONV, PHI, NSS and Maximum χ2) only suggested a total of 55 genes with a history of recombination. Detection of recombination in aligned sequences is quite variable [2] and the relative lack of sequence variation in *C. trachomatis *may have dampened trace detection thresholds.

Ninety-two genes showed significant evidence of positive selection. Of these, 23 genes showed overwhelming evidence of both positive selection and recombination. Studies in other pathogens [1,21] have also found recombination and positive selection occurring in tandem, especially in genomic regions coding for proteins involved in pathogenicity. Recombination may be effective in fixing beneficial mutations and removing deleterious ones, thus allowing a faster increase of fitness in the population under a wide range of conditions [14]. These 23 genes could represent situations where gene variants advantageous for *C. trachomatis *infection have been transmitted to other members of the species through gene conversion. A number of these genes have already been found in previous studies in *C. trachomatis *to have unusual levels of SNPs and genomic variation. *ompA *has been shown to be under selective immune and antibiotic pressure [32,67-69], which is consistent with the fact that it encodes MOMP, the immunodominant and most abundant protein of the elementary body (EB), the infectious form of *Chlamydia*. In addition, intragenic recombination was discovered within *ompA *[32,69] involving T cell epitopes and also in the regions immediately flanking this gene that were statistically shown to be hotspots for recombination [38]. The *Pmp *family, including *PmpE *and *PmpF*, are considered outer membrane proteins that play a role in antigenic variation and host immunity [70-72]. Indeed, *PmpF *was recently found to contain a high number of MHC class II epitopes in a region with significant amino acid variation among strains, indicating that the protein is likely under immune selection [73]. TARP is translocated into the host cell during EB attachment and is essential for cytoskeletal rearrangement that allows both entry of the organism and containment of the EB within an inclusion for subsequent replication [74]. Genetic variation within *tarp *(CT456), the gene that encodes TARP, tends to be similar for strains that are associated with the same disease phenotype, suggesting that the protein may be involved in niche-specific host adaptation [75]. Other important genes worth mentioning include *incD *(CT115; involved in development of the nascent *C. trachomatis *inclusion required for replication [76]), DNA-directed RNA polymerase subunit beta (*rpoB*; responsible for antibiotic resistance in many bacteria including *C. trachomatis *[56]) and heat shock protein 60 (groEL_2; a chaperon that is exposed on the cell surface and released by the cell during stress that may be involved in tissue pathology [77-79] and autoimmunity [35,43] While less is known about the role in virulence of some of the other genes (especially the hypothetical protein genes) revealed to have a history of positive selection and recombination, our screen suggests they may be important.

We are limited in our ability to discover recent recombination events by having only 12 completed genomes. However, we recently discovered an LGV strain that is a recombinant with strain D that involves *tarp *and other genes important in organism development and survival within the host cell [43]. Other genes with strong evidence from non-genome based screens that we did not identify in this study include the histone-H1-like protein gene (*hctB*; CT046), tryptophan synthase alpha chain gene (*trpA*; CT171), CTP synthtase (*pyrG*, CT183), hypothetical protein (CT360) and toxin gene (CT456) as having undergone homologous recombination [10,32,36-38,42].

*Chlamydia *grows in small intracellular populations and constantly goes through bottlenecks where a few bacteria transmit from host to host. This would seem to make the species highly susceptible to the effect of Muller's ratchet [3,80], an evolutionary process by which deleterious mutations accumulate in asexual populations. Moreover, in small populations like *C. trachomatis*, there are high chances for the occurrence of Hill-Robertson effect (HRE) [81], which imposes an important limit on the response to selection on the whole genome of asexuals by reducing the probability of fixation of beneficial alleles [82]. However, phylogenetic reconstructions show this group of organisms has survived for billions of years [83]. All these disruptive evolutionary processes can be limited by the effect of recombination, which allows beneficial mutations having evolved separately to come together [84]. Our results are further evidence that the intracellular niche of the bacterium is obviously not a barrier to exchange since the LD distance decay profile of *C. trachomatis *was similar to the free-lining pathogen/commensal *E. coli *(Figure 5).

One of the major factors that make recombination more likely to occur between members of clades within a bacterial species is frequent physical proximity [2]. This proximity is more likely to occur between members of the same community, which are likely to be related because many species of bacteria show significant spatial [85] and ecological [86] structuring. For *C. trachomatis *to recombine regularly there must be frequent mixed infections with sufficient organisms to allow individual cells to be invaded. We have very little understanding of the nature and frequency of infections where *C. trachomatis *DNA exchange can take place, although we know that repeat infection occurs at rates as high as 56.9% [43,87,88] and mixed infections at rates of up to 15% [67,89,90] and probably higher. Our reconstruction of inter-clade recombination currents (Figure 4 & Table 5) indicates that there has historically been a certain level of sexual isolation between clades 1 and 2 on the one hand and clades 3 and 4 on the other (although we have recently discovered clear evidence of very recent gene conversion from a serovar D to L_2 _from men having sex with men (MSM) [43]). These patterns suggest a spatial structure in *C. trachomatis *infections, with some strains associated with a particular tissue or category of host. Some evidence points in that direction, for example the G strains have been noted to be more prevalent in rectal tissues in MSM while E strains are more prevalent in the cervix among populations in the same geographical region [91-93]. In such spatially structured environment, it has been predicted that if beneficial mutations (due to selection) are high, adaptation proceeds more slowly mainly because of Hill-Robertson effect [94]. This will develop negative associations among favored alleles containing those beneficial mutations, which favors an increased rate in recombination in order to fix those beneficial mutations in the population. In other words, the adaptive cost of the spatial structuring in *C. trachomatis *strains is compensated by recombination, which might explain the observed currents of recombination flux.

## Conclusions

In this study, we evaluated the contribution of recombination and positive selection in *C. trachomatis *based on comparative analysis of 12 completed published genomes. Even though the gene content of the 12 strains was very similar, phylogenetic reconstruction using the ClonalFrame software suggested that a steady rate of recombination has contributed significantly towards the genetic diversity of *C. trachomatis *(r/m mean estimate of 0.71). We observed trends of distance-dependent decay of linkage disequilibrium in *C. trachomatis *core genes, similar to other recombining lineages of bacteria such as *E. coli*. We identified seven genes known to be involved in the virulence and pathogenicity of infection with a history of both positive selection and invasion of *C. trachomatis *lineages through gene conversion. Sixteen other *C. trachomatis *genes have the same evolutionary dynamics, suggesting they should be targeted for further studies of molecular pathogenesis. In tracing the origins of the imports of recombinant fragments we observed more DNA exchange between clades 1 and 2 and between clades 3 and 4, which could indicate historical spatial population structure in *C. trachomatis *infections corroborating with its tissue specific infection in the host cells, intra-cellular nature of infection as well as nature (mixed infection) and frequency of infection. All these factors facilitate the occurrence of recombination and positive selection to adapt to the varying host and external environments.

## Competing interests

The authors declare that they have no competing interests.

## Authors' contributions

SJJ, TDR and DD conceptualized the study. SJJ conducted the OrthoMCL analyses, phylogenetic analyses, analysis for positive selection, analyses for intragenic recombination and calculation of linkage disequilibrium. XD and SJJ performed the ClonalFrame analysis. KG, SJJ and XD processed the output of ClonalFrame. SJJ wrote the manuscript. TDR, XD and DD contributed to the writing of the manuscript. TDR and DD provided oversight to all the analyses. All authors read and approved the final manuscript.

## Reviewer's Comments

### Referee 1: Dr Jeremy Selengut

This work provides a thorough assessment of evolutionary processes in the *Chlamydia trachomatis *lineage from our current vantage of 12 complete genomes. The methods applied in the determination of the relative frequency and importance of mutation and recombination events are proper and have been used to dissect the history of the 92 genes showing evidence of positive

selection. The authors interpret their data as supporting a model of mutation, inter-strain lateral gene transfer and gene conversion via recombination and positive selection through gene loss of the original gene in the recipient strain. That genes falling into this class are enriched in known virulence factors leads them to the intriguing suggestion that other genes of unknown function with the same patterns of mutational distribution may have a similar role. The discussion of the interplay between the forces of genetic degradation in asexual organisms characterized by bottlenecking and the rescue of beneficial mutations through recombination provides the necessary context for understanding the extent to which these observations can be directly applied to other lineages.

#### Author's response

We completely agree with the reviewer's suggestion that comparative genome analysis as well as in silico methods we implemented in this study can be effectively used to understand and dissect the evolutionary forces acting on other microbial lineages that survived for millions of years. The impact of genomics and such computational methods will be peaked for microbial lineages that invade the host at intracellular level and those that lack a genetic system to understand more about the evolutionary history of such lineages.

### Referee 2: Dr I. King Jordan

This article was reviewed by **Lee S. Katz**, Meningitis and Vaccine Preventable Diseases Branch, Centers for Disease Control and Prevention, Atlanta, GA USA (nominated by I. King Jordan)

This paper uses several statistical methods to uncover and characterize recombination events in the history of *C. trachomatis*. The authors uncover an impressive wealth of information from 12 genomes (plus an outgroup). The methods in this paper are sound and well reasoned. I have no major points.

### Minor points

1) Alignments and Phylogenetic Inference, last sentence: It is not clear what "cf" stands for.

#### Author's response

*We apologize for this mistake that occurred while editing. This has now been removed from the manuscript*.

2) Evolutionary analysis using ClonalFrame, second paragraph: "Highest normalized BLASTN score." The evalue is a probabilistic representation of the normalized score. It is not clear why the authors chose to use a custom normalized score rather than the e-value that BLASTN already produces.

#### Author's response

*Both methods for processing the BLAST results arrive at the same result*. We used the normalized score [95] only because we had used it in previous analyses of Chlamydia genomes [96].

3) Calculation of Linkage Disequilibrium, last sentence: I think it would make the paper stronger if the authors included the formula for the DA' metric and an explanation of DA'.

#### Author's response

*The formula for estimating D*'_*A *_*metric along with a short explanation of the method has been added to the manuscript on page 10.*

4) The elementary body mentioned on page 15 is described as a particle. However, for the purposes of clarity it may help to describe it as a stage in the *C. trachomatis *lifecycle. There are two major phases in he lifecycle: the noninfectious reticulate body (RB) and the infectious and intracellular elementary body (EB). It is confusing when the cell is instead described as a particle.

#### Author's response

*The text has been changed to remove the word 'particle'*.

5) Figure 1 legend. It may help the reader if you label NG as the out-group, even though it is labeled in Table 1.

#### Author's response

*Figure *1 is modified and we labeled Ng as the out-group.

6) Figure 1. It is unclear what the units are on the scale. Is it amino acid residues?

#### Author's response

*We generated figure *1 using the TreeView software. In TreeView the scale bar usually indicates the branch length. In this study, we used PHYLIP's CONSENSE program for inferring the species tree, which instead of showing branch lengths, shows the bootstrap values in the nexus tree output file. Because PHYLIP puts these numbers where TreeView expects the branch lengths, TreeView will display a scale bar, which in our case is an error scale bar.

We edited figure 1 and removed the scale bar. We thank the reviewer for noticing this.

7) Table 1. It may help if the table also includes genome size and/or number of genes per 1 kb so that the reader has a better grasp of the *C. trachomatis *genome.

#### Author's response

*We added the genome size of each of the C. trachomatis strains into table *1.

### Very minor points

1) Background, paragraph 2: "mutations" should be singular

#### Author's response

This mistake is now corrected in the manuscript.

2) Evidence for Positive Selection, last sentence: The p-value should be "less than or equal to" instead of "equal to."

#### Author's response

*We edited the manuscript accordingly*.

3) Italicize pyrG at the end of page 16 because it is a gene name.

#### Author's response

We edited the manuscript accordingly

4) Figure 3: the resolution is poor. It would help the quality of the paper if the image were saved as a PNG or SVG instead of a JPG (which is a better format for photographs).

#### Author's response

*We made figure *3 as a PNG image with improved resolution and quality.

5) Figure 4. It may help emphasize the authors' stance on the one-sided flow of genes between clades if the arrows were weighted differently according to the number of genes.

#### Author's response

Thanks for the nice suggestion. We increased the width of the connecting lines and used different colors to highlight the clade-specific gene flow we identified in this study.

### Referee 3: Dr Arcady Mushegian

This is a useful comparative study of many isolates of *Chlamydia trachomatis*. I have mostly technical questions and comments:

1) p. 7: "OrthoMCL was run with a BLAST E-value cut-off of 1e-05." -- was it run against the NR database, against all species minus the query species, or against each species separately? The E-value would be different for the same HSP in these three situations, as it is computed using the database length and is reverse proportional to it (whereas the score S would be the same). If the searches were done against the small database of a single proteome, the E-value of e-05 is probably too restrictive, and the list of "core genes" is longer. As a quick test of this, were all short ribosomal proteins (which are sure to be omnipresent in these genomes) reported among the orthologous genes? What would change if the E-value was set at e-02 or e-03 - would the "core gene" list cover closer to the 100% of the annotated genes in the smallest genome? And would the properties of these genes be the same or affect the conclusions?

#### Author's response

The blastp was not run against the NR database or against all the strains minus the query strain or against each species separately. Instead we performed all-against-all BLASTP comparison of all the protein sequences from the genomes used in this study. We created a blast database using all the protein sequences of the Chlamydia strains used in this study and performed blastp against it with the same dataset (all protein sequences).

2) p. 10: "It has been suggested that the majority of apparent disparity in gene content might be caused by differences in the gene predictions rather than being real differences in the presence or absence of genes" - a quick way to estimate the possible effect of this is to look for the homologs of "unique" or sparsely present genes in the other genomes by searching the nucleotide sequence with the TBLASTN program. Has this been attempted, can it also make the list of "core genes" longer, and would these genes be different in any way (other than they would perhaps be shorter-than-median gene length)?

#### Author's response

We took all unique or sparsely present gene clusters and performed TBLASTN analysis against a database containing all the genomes of Chlamydia strains used in this study. We found that 15 such gene clusters had perfect matches to all of the 12 genomes in the BLAST database, which increases the core genome size from 836 to 851 genes. For this study we used the annotation provided through Genbank but this point illustrates that we need to consider re-annotating consistently for future multi-genome comparisons.

3) p. 11: "four-substitution" is a typo, change to "four substitution", and the same for "four-recombination"?

#### Author's response

We corrected the manuscript accordingly.

4) p. 13: "The 95% credibility interval of r/m was 0.56-1.01 (mean = 0.71), indicating that unlike mutation recombination often affects several nucleotides at each occurrence." -- probably, awkward wording: does not recombination affect more than one base by definition?

#### Author's response

We agree with the reviewer's comment and corrected the sentence by replacing the word 'affects' with 'substitutes'.

p. 14: "more inter-clade DNA exchange between clades 1 and 2 and between clades 3 and 4" -- if the genes involved in these exchanges are excluded from the concatenated alignment used to build the genome tree, would the clades remain the same? In other words, is HGT determining the topology (or at least the statistical support for the partitions) of the tree?

#### Author's response

We redid the phylogenetic analysis by excluding the genes involved in the inter-clade DNA exchange. We noticed that there were neither rearrangements of the clades nor any significant changes in the statistical support for the partitions of the clades in the new species tree.

## Supplementary Material

Additional file 1 **Gene loci identified as being under intragenic homologous recombination using the 4 methods of Substitution analysis of recombination (p-value < 0.05)**. Click here for file

Additional file 2 **Gene loci under positive selection inferred based on the overall test (Test 1)**. Click here for file

Additional file 3 **Gene loci under positive selection based on the branch-site specific test (Test 2)**. Click here for file

Additional file 4 **Inter-clade events inferred by ClonalFrame grouped according to the affected clade and the clade of origin**. Click here for file
