# Supplementary material for: Interplay of recombination and selection in the genomes of Chlamydia trachomatis
Source: Biol Direct. 2011 May 26;6:28. doi: 10.1186/1745-6150-6-28 (PMC3126793; doi:10.1186/1745-6150-6-28)
Supplement: Additional file 1 — Gene loci identified as being under intragenic homologous recombination using the 4 methods of Substitution analysis of recombination (p-value < 0.05). [file 1745-6150-6-28-S1.PDF]

Additional File 1. Gene loci identified as being under intragenic homologous recombination using the 4 methods of Substitution analysis of recombination (p-value < 0.05).

**GENECONV**			
**Gene locus Tag**	**locus**	**Annotation**	**p-value**
CT643	*topA*	DNA topoisomerase I/SWI domain fusion protein	0
CT852	*yhgN*	YhgN family protein/putative integral membrane protein	0
CT652	*recD_2*	exodeoxyribonuclease V alpha chain	0
CT869	*pmpE*	polymorphic outer membrane protein	0
CT870	*pmpF*	polymorphic outer membrane protein	0
CT872	*pmpH*	polymorphic outer membrane protein	0
CT011	-	hypothetical protein	0
CT049	-	hypothetical protein	0
CT144	-	hypothetical protein	0
CT244	-	hypothetical protein	0
CT674	*yscC*	Yop proteins translocation protein C/general secretion pathway protein/Type III secretion structural protein (outer membrane ring)	0
CT678	*pyrH*	uridylate kinase	0
CT679	*tsf*	elongation factor Ts	0
CT681	*ompA*	major outer membrane protein	0
CT682	*pbpB*	penicillin-binding protein	0
CT402	*lpxK*	tetraacyldisaccharide 4'-kinase	0
CT448	*secD/secF*	bifunctional preprotein translocase subunit SecD/SecF	0
CT604	*GroEL_2*	HSP-60/60 kDa chaperonin GroEL2	0
CT619	-	hypothetical protein	0
CT107	*mutY*	A/G-specific adenine glycosylase	0.0003
CT456	-	Hypothetical protein/Translocated actin-recruiting phosphoprotein (tarp protein)	0.0004
CT675	*karG*	ATP:guanido phosphotransferase	0.0011
CT676	-	hypothetical protein	0.0015
CT653	*yhbG*	ABC transporter ATP-binding protein	0.0046
CT112	*pepF*	oligoendopeptidase F	0.0127
CT685	-	ABC transporter ATP-binding protein	0.0135
CT052	*hemN_1*	coproporphyrinogen III oxidase	0.0332
CT873	-	hypothetical protein	0.0425
**Maximum $\chi 2$**			
**Gene**	**locus**	**Annotation**	**p-value**

**locus Tag**			
CT674	*yscC*	Type III secretion structural protein (outer membrane ring)	0.00E+00
CT681	*ompA*	major outer membrane protein	0.00E+00
CT869	*pmpE*	polymorphic outer membrane protein	0.00E+00
CT870	*pmpF*	polymorphic outer membrane protein	0.00E+00
CT872	*pmpH*	polymorphic outer membrane protein	0.00E+00
CT049	-	hypothetical protein	0.00E+00
CT144	-	hypothetical protein	0.00E+00
CT456	-	Translocated actin-recruiting phosphoprotein (tarp protein)	0.00E+00
CT619	-	hypothetical protein	0.00E+00
CT679	*tsf*	elongation factor Ts	0.00E+00
CT871	*pmpG*	polymorphic outer membrane protein	0.00E+00
CT640	*recC*	exodeoxyribonuclease V gamma chain	0.00E+00
CT682	*pbpB*	penicillin-binding protein	0.00E+00
CT448	*secD/secF*	bifunctional preprotein translocase subunit SecD/SecF	0.00E+00
CT011	-	hypothetical protein	0.00E+00
CT244	-	hypothetical protein	0.00E+00
CT402	*lpxK*	tetraacyldisaccharide 4'-kinase	0.00E+00
CT604	*GroEL_2*	60 kDa chaperonin GroEL2	0.00E+00
CT680	*rpsB*	30S ribosomal protein S2	1.00E-03
CT653	*yhbG*	ABC transporter ATP-binding protein	1.00E-03
CT852	*yhgN*	YhgN family protein/putative integral membrane protein	1.00E-03
CT675	*karG*	ATP:guanido phosphotransferase	2.00E-03
CT652	*recD_2*	exodeoxyribonuclease V alpha chain	2.00E-03
CT684	-	cysteine desulfurase activator complex subunit SufB	2.00E-03
CT678	*pyrH*	uridylate kinase	3.00E-03
CT676	-	hypothetical protein	4.00E-03
CT470	*recO*	DNA repair protein RecO	4.00E-03
CT621	-	hypothetical protein	4.00E-03
CT875	-	hypothetical protein	8.00E-03
CT677	*frrr*	ribosome recycling factor	8.00E-03
CT295	*mrsA_1*	phosphoglucomutase	8.00E-03
CT686	-	hypothetical protein	9.00E-03
CT115	-	inclusion membrane protein D	1.10E-02
CT580	-	Hypothetical protein/putative integral membrane protein	1.10E-02
CT642	-	hypothetical protein	1.20E-02
CT685	-	ABC transporter ATP-binding protein	1.30E-02

CT683	-	TPR-motif-containing protein/tetratricopeptide repeat protein	1.30E-02
CT688	*parB*	putative chromosome partitioning protein	1.40E-02
CT107	*mutY*	A/G-specific adenine DNA glycosylase	1.90E-02
CT727	*zntA*	metal transport P-type ATPase/cation transporting ATPase	1.90E-02
CT551	*dacC*	D-alanyl-D-alanine carboxypeptidase	2.00E-02
CT315	*rpoB*	DNA-directed RNA polymerase subunit beta	2.10E-02
CT650	*recA*	recombinase A	2.20E-02
CT145	-	serine/threonine-protein kinase PKN1	2.70E-02
CT643	*topA*	DNA topoisomerase I/SWI domain fusion protein	3.00E-02
CT020	*lepB*	signal peptidase I	4.00E-02
CT874	*pmpI*	polymorphic outer membrane protein	4.60E-02
**Pairwise Homoplasy Index (PHI)**			
**Gene locus Tag**	**locus**	**Annotation**	**p-value**
CT680	*rpsB*	30S ribosomal protein S2	0.00E+00
CT681	*ompA*	major outer membrane protein	0.00E+00
CT869	*pmpE*	polymorphic outer membrane protein	0.00E+00
CT870	*pmpF*	polymorphic outer membrane protein	0.00E+00
CT872	*pmpH*	polymorphic outer membrane protein	0.00E+00
CT049	-	hypothetical protein	0.00E+00
CT144	-	hypothetical protein	0.00E+00
CT679	*tsf*	elongation factor Ts	1.00E-02
CT824	-	insulinase family metalloprotease	1.70E-02
CT676	-	hypothetical protein	2.90E-02
CT677	*frrr*	ribosome recycling factor	3.40E-02
CT674	*yscC*	Type III secretion structural protein	4.30E-02
CT686	-	ABC transporter membrane protein	4.80E-02
**Neighbor Similarity Score (NSS)**			
**Gene locus Tag**	**locus**	**Annotation**	**p-value**
CT674	*yscC*	Type III secretion structural protein	0.00E+00
CT675	*karG*	ATP:guanido phosphotransferase	0.00E+00
CT680	*rpsB*	30S ribosomal protein S2	0.00E+00

CT681	*ompA*	major outer membrane protein	0.00E+00
CT686	-	ABC transporter membrane protein	0.00E+00
CT869	*pmpE*	polymorphic outer membrane protein	0.00E+00
CT870	*pmpF*	polymorphic outer membrane protein	0.00E+00
CT872	*pmpH*	polymorphic outer membrane protein	0.00E+00
CT049	-	hypothetical protein	0.00E+00
CT144	-	hypothetical protein	0.00E+00
CT824	-	metalloprotease-insulinase	5.00E-03
CT288	-	Hypothetical protein/candidate inclusion membrane protein	5.00E-03
CT838	-	hypothetical protein/putative membrane transport protein	6.00E-03
CT456	-	Translocated actin-recruiting phosphoprotein (Tarp)	7.00E-03
CT619	-	hypothetical protein	7.00E-03
CT700	-	hypothetical protein	9.00E-03
CT679	*tsf*	elongation factor Ts	1.60E-02
CT871	*pmpG*	polymorphic outer membrane protein	1.70E-02
CT640	*recC*	exodeoxyribonuclease V gamma chain	3.20E-02
CT682	*pbpB*	penicillin-binding protein	4.40E-02
CT033	*recD_1*	exodeoxyribonuclease V alpha chain	4.40E-02
