# Supplementary material for: Interplay of recombination and selection in the genomes of Chlamydia trachomatis
Source: Biol Direct. 2011 May 26;6:28. doi: 10.1186/1745-6150-6-28 (PMC3126793; doi:10.1186/1745-6150-6-28)
Supplement: Additional file 2 — Gene loci under positive selection inferred based on the overall test (Test 1). [file 1745-6150-6-28-S2.PDF]

Additional File 2. Gene loci under positive selection inferred based on the overall test (Test 1)

Gene Locus_tag	Locus	Annotation	LRT -p-value	FDR-p-value
CT681	*ompA*	major outer membrane protein	0.000116125	0.002606405
CT694	-	hypothetical protein	0.005119211	0.06248935
CT711	-	hypothetical protein	0.008126101	0.09119444
CT745	*hemG*	protoporphyrinogen oxidase	0.001364036	0.02109071
CT774	*cysQ*	3'(2'),5'-bisphosphate nucleotidase	0.003787879	0.04972767
CT809	-	hypothetical protein	0.00467214	0.06020048
CT823	*htrA*	serine protease	4.17E-06	0.000145072
CT824	-	insulinase family zinc metalloprotease	2.68E-06	9.81E-05
CT826	*pssA*	CDP-diacylglycerol--serine O-phosphatidyltransferase	0.00148357	0.02244027
CT840	*mesJ*	tRNA(Ile)-lysidine synthase/ PP-loop superfamily ATPase	0.002079681	0.0291408
CT841	*ftsH*	ATP-dependent zinc protease/Cell division protein	0.004771383	0.06036142
CT847	-	hypothetical protein	0.002321161	0.03166744
CT859	*ispH*	4-hydroxy-3-methylbut-2-enyl diphosphate reductase	0.001348244	0.02109071
CT860	-	hypothetical protein	0.004925704	0.06120095
CT866	*glgB*	glycogen branching enzyme	1.25E-06	4.83E-05
CT867	-	hypothetical protein	1.82E-07	8.44E-06
CT868	-	hypothetical protein	4.48E-07	1.89E-05
CT869	*pmpE*	polymorphic outer membrane protein	4.45E-35	1.55E-32
CT870	*pmpF*	polymorphic outer membrane protein	3.05E-35	1.55E-32
CT875	-	hypothetical protein	3.37E-09	2.93E-07
CT011	-	hypothetical protein	0.000466488	0.008772363
CT012	-	Hypothetical protein/putative integral membrane protein	1.84E-14	2.56E-12
CT013	*cydA*	cytochrome D ubiquinol oxidase subunit I	0.003609565	0.04829803
CT015	*phoH*	ATPase/hypothetical protein	8.84E-05	0.002113776
CT017	-	hypothetical protein	0.007273621	0.08434848
CT018	-	hypothetical protein	0.000540329	0.009639878
CT040	*ruvB*	Holliday junction DNA helicase RuvB	0.000172746	0.003642268
CT042	*gigX*	glycogen hydrolase	0.000479851	0.008786191
CT049	-	hypothetical protein	1.20E-07	6.42E-06
CT056	-	hypothetical protein	0.007953859	0.09072478
CT064	*lepA*	GTP-binding protein LepA	0.000171077	0.003642268
CT082	-	hypothetical protein	4.61E-07	1.89E-05
CT089	*icrE*	low calcium response protein E (TTSS effector protein)	3.80E-05	0.001100994
CT099	*trxB*	thioredoxin reductase	0.001601461	0.02370808

CT107	*mutY*	A/G-specific adenine DNA glycosylase	2.10E-12	2.44E-10
CT109	-	hypothetical protein	6.07E-05	0.001632857
CT115	-	inclusion membrane protein D	1.36E-05	0.000451064
CT139	*oppA_1*	oligopeptide transport system binding protein	0.000878121	0.01490213
CT140	-	Hypothetical protein/exported protein	0.001781622	0.0258257
CT144	-	hypothetical protein	3.32E-15	5.78E-13
CT147	-	Hypothetical protein/putative integral membrane protein	2.72E-16	6.31E-14
CT198	*oppA_3*	oligopeptide transport system binding protein	0.002094082	0.0291408
CT205	*pfkA_1*	diphosphate--fructose-6-phosphate 1-phosphotransferase	1.19E-08	9.20E-07
CT209	*leuS*	leucyl-tRNA synthetase	1.48E-07	7.36E-06
CT211	-	hypothetical protein	6.10E-05	0.001632857
CT212	-	hypothetical protein	8.48E-05	0.002106175
CT213	*rpiA*	ribose-5-phosphate isomerase A	0.000598795	0.01041588
CT216	*xasA*	glutamate/gamma-aminobutyrate antiporter	0.006532079	0.07703308
CT223	-	hypothetical protein	4.90E-08	2.95E-06
CT227	-	hypothetical protein	6.59E-05	0.00169819
CT229	-	hypothetical protein	2.86E-05	0.000864391
CT244	-	hypothetical protein	4.55E-08	2.95E-06
CT249	-	hypothetical protein	0.000193596	0.00384863
CT286	*clpC*	ATP-dependent Clp protease	0.001214478	0.01965165
CT288	-	Hypothetical protein/candidate inclusion membrane protein	5.09E-08	2.95E-06
CT362	*lysC*	aspartate kinase	0.005246584	0.06293996
CT455	*murA*	UDP-N-acetylglucosamine 1-carboxyvinyltransferase	1.95E-05	0.000615701
CT456	-	hypothetical protein/translocated actin-recruiting protein (tarp)	9.11E-05	0.002113776
CT470	*recO*	DNA repair protein RecO	0.000180022	0.003684042
CT529	-	hypothetical protein	0.001031138	0.01708226
CT580	-	hypothetical protein/putative integral membrane protein	1.47E-11	1.46E-09
CT604	*groEL_2*	60 kDa chaperonin GroEL2	0.000333033	0.00643669
