# Supplementary material for: Interplay of recombination and selection in the genomes of Chlamydia trachomatis
Source: Biol Direct. 2011 May 26;6:28. doi: 10.1186/1745-6150-6-28 (PMC3126793; doi:10.1186/1745-6150-6-28)
Supplement: Additional file 3 — Gene loci under positive selection based on the branch-site specific test (Test 2). [file 1745-6150-6-28-S3.PDF]

Additional File 3. Gene loci under positive selection based on the branch-site specific test (Test 2).

Gene Locus_tag	Locus	Annotation	LRT p-value	FDR p-value
**Clade 1**				
**Gene Locus_tag**	**Locus**	**Annotation**	**LRT p-value**	**FDR p-value**
CT852	*yhgN*	Yhgn Family Protein/putative integral membrane protein	0.000141547	0.01888237
CT868	-	hypothetical protein	2.45E-05	0.005447167
CT115	-	inclusion membrane protein D	1.07E-06	0.000356845
CT147	-	Hypothetical protein/putative integral membrane protein	0.000174239	0.01936957
CT249	-	hypothetical protein	0.000668414	0.07810895
CT456	-	hypothetical protein /Translocated actin-recruiting phosphoprotein	9.45E-09	6.30E-06
CT619	-	hypothetical protein	4.92E-05	0.0082041
**Clade 2**				
**Gene Locus_tag**	**Locus**	**Annotation**	**LRT -p-value**	**FDR-p-value**
CT011	-	hypothetical protein	3.28E-06	0.002523685
CT308	*atpA*	V-type ATP synthase subunit A	0.000333876	0.07953066
CT341	*dnaJ*	heat shock protein J /chaperone protein DnaJ	0.000388903	0.07953066
CT380	*phnP*	metal-dependent hydrolase	6.17E-06	0.002523685
**Clade 3**				
**Gene Locus_tag**	**Locus**	**Annotation**	**LRT -p-value**	**FDR-p-value**
CT823	*htrA*	DO Serine protease	0.000103724	0.007713294
CT827	*nrdA*	ribonucleotide-diphosphate reductase subunit alpha	0.00169824	0.07717557
CT870	*pmpF*	polymorphic outer membrane protein	0.000673141	0.03670862
CT012	-	hypothetical protein	5.19E-26	2.12E-23
CT065	-	ADP,ATP carrier protein	6.31E-05	0.005162782
CT082	-	hypothetical protein	2.98E-06	0.000305145
CT115	-	inclusion membrane protein D	0.001202199	0.06146242
CT147	-	Hypothetical protein/putative integral membrane protein	3.59E-17	7.34E-15
CT209	*leuS*	leucyl-tRNA synthetase	0.000150662	0.01027013
CT227	-	hypothetical protein	1.20E-09	1.63E-07
CT244	-	hypothetical protein	9.54E-11	1.56E-08
CT254	-	hypothetical protein/inner membrane protein	0.00042992	0.02705189
CT315	*rpoB*	DNA-directed RNA polymerase subunit beta	0.001389792	0.06687352
CT375	-	putative oxidoreductase	0.00054153	0.03164082
CT402	*ipxK*	tetraacyldisaccharide 4'-kinase	2.49E-26	2.04E-23
CT448	*secD/secF*	bifunctional preprotein translocase	2.14E-07	2.50E-05

CT448	*secD/secF*	bifunctional preprotein translocase subunit SecD/SecF	2.14E-07	2.50E-05
CT591	*sdhB*	succinate dehydrogenase iron-sulfur subunit	1.80E-05	0.001632655
CT604	*GroEL_2*	HSP 60/60 kDa chaperonin GroEL2	4.95E-18	1.35E-15
**Clade 4**				
**Gene Locus_tag**	**Locus**	**Annotation**	**LRT p-value**	**FDR p-value**
CT707	*tig*	trigger factor	0.001668657	0.04300546
CT739	*ftsK*	cell division protein FtsK	0.000938103	0.0316283
CT745	*hemG*	protoporphyrinogen oxidase	1.31E-09	8.32E-07
CT748	*mfd*	transcription-repair coupling factor	0.000859936	0.0316283
CT756	*murF*	UDP-N-acetylmuramoyl-tripeptide--D-alanyl-D-alanine ligase	0.001175782	0.03354957
CT762	*murC/ddlA*	bifunctional D-alanyl-alanine synthetase A/UDP-N-acetylmuramate--L-alanine ligase	0.005000301	0.09987443
CT768	-	hypothetical protein	0.000459232	0.02616963
CT779	-	hypothetical protein	0.000716955	0.0316283
CT782	*cysS*	cysteinyl-tRNA synthetase	0.001021935	0.03265889
		glycyl-tRNA synthetase	0.000495571	0.02616963
CT840	*mesJ*	PP-loop superfamily ATPase/tRNA(Ile)-lysidine synthase	0.001320635	0.03638337
CT841	*ftsH*	ATP-dependent zinc protease/Cell division protein	0.002214862	0.0877202
CT847	-	hypothetical protein	0.000284971	0.01805816
CT875	-	hypothetical protein	3.71E-06	0.001176546
CT033	*recD_1*	exodeoxyribonuclease V alpha chain	0.000709905	0.0316283
CT108	*ybgI*	Acr family transporter	0.00213236	0.05162556
CT112	*pepF*	oligoendopeptidase F	0.000180174	0.01427168
CT114	-	hypothetical protein	0.000802599	0.0316283
CT149	-	hydrolase	0.003421852	0.07388864
CT223	-	hypothetical protein	0.001141791	0.05168107
CT249	-	hypothetical protein	0.003250097	0.07212935
CT286	*clpC*	ATP-dependent ClpC protease	0.000932156	0.0316283
CT288	-	Hypothetical protein/candidate inclusion membrane protein	2.76E-05	0.005837499
CT289	-	hypothetical protein	0.000116624	0.01055754
CT301	*pknD*	serine/threonine-protein kinase	0.004700505	0.09629375
CT305	*atp1*	V-type ATP synthase subunit I	0.000281513	0.01805816
CT306	*atpD*	V-type ATP synthase subunit D	0.001136536	0.03354957
CT315	*rpoB*	DNA-directed RNA polymerase subunit beta	5.83E-05	0.006627397
CT317	*rplJ*	50S ribosomal protein L10	0.001714316	0.07242233
CT327	*trpC*	N-(5'-phosphoribosyl)anthranilate [illegible]	[illegible]	[illegible]

CT329	*xseA*	exodeoxyribonuclease VII large subunit	4.88E-05	0.006627397
CT379	*hflX*	GTP binding protein	0.001448642	0.03857962
CT397	*vacB*	exoribonuclease II	0.004524844	0.09513453
CT477	*ada*	methylated-DNA protein - cysteine methyltransferase	0.000924583	0.0316283
CT478	*oppC_2*	oligonucleotide transport system permease	6.28E-05	0.006627397
CT479	*oppB_2*	oligopeptide permease	0.003219834	0.07212935
CT480	*oppA_4*	oligopeptide transport system, binding lipoprotein	0.000950099	0.0316283
CT500	*ndk*	nucleoside diphosphate kinase	0.00107037	0.03289113
CT551	*dacC*	D-alanyl-D-alanine carboxypeptidase	0.001074055	0.05168107
CT609	*rpoN*	RNA polymerase factor sigma-54	0.001811551	0.04522918
