# Supplementary material for: Interplay of recombination and selection in the genomes of Chlamydia trachomatis
Source: Biol Direct. 2011 May 26;6:28. doi: 10.1186/1745-6150-6-28 (PMC3126793; doi:10.1186/1745-6150-6-28)
Supplement: Additional file 4 — Inter-clade events inferred by ClonalFrame grouped according to the affected clade and the clade of origin. [file 1745-6150-6-28-S4.PDF]

Additional File 4. Inter-clade events inferred by ClonalFrame grouped according to the affected clade and the clade of origin.

**CLADE 1-> CLADE 2**				
**Recombination Event ID**	**Start**	**End**	**Width of the import (bp)**	**Putative Origin (clade) of the import**
Clade1_L2b00007	486521	486905	384	2
Clade1_L2b00019	545115	545230	115	2
Clade1_L2b00034	648664	648721	57	2
Clade1_L2b00035	648833	648961	128	2
Clade1_L2b00036	826917	827016	99	2
Clade1_L2b00040	928424	928818	394	2
Clade1_L2b00058	11312	11764	452	2
Clade1_L2b00073	107201	107339	138	2
Clade1_L2b00083	111456	112132	676	2
Clade1_L2b00087	155762	155882	120	2

**CLADE 1 -> CLADE 3**				
**Recombination Event ID**	**Start**	**End**	**Width of the import (bp)**	**Putative Origin (clade) of the import**
Clade1_L2b00008	491513	491609	96	3
Clade1_L2b00013	535222	535322	100	3
Clade1_L2b00026	323656	323877	221	3
Clade1_L2b00070	89143	89466	323	3

**CLADE 1 -> CLADE 4**				
**Recombination Event ID**	**Start**	**End**	**Width of the import (bp)**	**Putative Origin (clade) of the import**
Clade1_L2b00009	493999	494171	172	4
Clade1_L2b00022	452665	453350	685	4
Clade1_L2b00060	15573	15918	345	4
Clade1_L2b00068	86736	87346	610	4

**CLADE 2 -> CLADE 1**				
**Recombination Event ID**	**Start**	**End**	**Width of the import (bp)**	**Putative Origin (clade) of the import**
Clade2_E00002	2474	3423	949	1
Clade2_E00003	175041	175144	103	1
Clade2_E00009	312635	313391	756	1
Clade2_E00012	315481	315724	243	1
Clade2_E00015	317016	317330	314	1
Clade2_E00026	118230	118368	138	1
Clade2_E00028	596334	596556	222	1
Clade2_E00040	905426	906408	982	1
Clade2_E00041	906039	906250	211	1

Clade2_E00045	1005017	1005412	395	1
Clade2_E00050	126230	126848	618	1
Clade2_E00052	161773	161941	168	1
Clade2_E00055	164250	164659	409	1
**CLADE 2 -> CLADE 3**				
**Recombination Event ID**	**Start**	**End**	**Width of the import (bp)**	**Putative Origin (clade) of the import**
Clade2_E00053	162475	163023	548	3
Clade2_E00054	163938	164172	234	3
**CLADE 2 -> CLADE 4**				
**Recombination Event ID**	**Start**	**End**	**Width of the import (bp)**	**Putative Origin (clade) of the import**
Clade2_E00005	225061	225796	735	4
Clade2_E00011	129095	129980	885	4
Clade2_E00023	326325	326925	600	4
Clade2_E00047	121297	121462	165	4
Clade2_E00048	122340	122451	111	4
**CLADE 3 -> CLADE 1**				
**Recombination Event ID**	**Start**	**End**	**Width of the import (bp)**	**Putative Origin (clade) of the import**
Clade3_BTz00039	994511	994690	179	1
**CLADE 3 -> CLADE 2**				
**Recombination Event ID**	**Start**	**End**	**Width of the import (bp)**	**Putative Origin (clade) of the import**
Clade3_BTz00023	622723	622773	50	2
Clade3_BTz00032	734907	734972	65	2
Clade3_BTz00044	171517	171727	210	2
**CLADE 3 -> CLADE 4**				
**Recombination Event ID**	**Start**	**End**	**Width of the import (bp)**	**Putative Origin (clade) of the import**
Clade3_BTz00007	314936	315672	736	4
Clade3_BTz00008	317162	317294	132	4
Clade3_BTz00009	317501	317627	126	4
Clade3_BTz00010	317876	318815	939	4
Clade3_BTz00011	164265	164917	652	4
Clade3_BTz00015	332245	332376	131	4
Clade3_BTz00016	332406	332542	136	4
Clade3_BTz00024	635643	636433	790	4
Clade3_BTz00027	675251	675392	141	4

Clade3_BTz00034	907202	907356	154	4
Clade3_BTz00050	183007	183130	123	4
**CLADE 4 -> CLADE 1**				
Recombination Event ID	Start	End	Width of RE (bp)	Putative Origin of RE
Clade4_00006	163360	164488	1128	1
**CLADE 4 -> CLADE 2**				
Recombination Event ID	Start	End	Width of RE (bp)	Putative Origin of RE
Clade4_00013	176191	176413	222	2
**CLADE 4 -> CLADE 3**				
Recombination Event ID	Start	End	Width of RE (bp)	Putative Origin of RE
Clade4_00017	312223	312928	705	3
Clade4_00018	312821	313579	758	3
Clade4_00021	314232	315312	1080	3
Clade4_00023	315543	315774	231	3
Clade4_00024	316605	316918	313	3
Clade4_00025	317286	318021	735	3
Clade4_00028	325333	325711	378	3
Clade4_00030	326206	326715	509	3
Clade4_00032	330157	330454	297	3
Clade4_00037	469300	469505	205	3
Clade4_00040	672175	672721	546	3
Clade4_00042	672549	672862	313	3
Clade4_00043	672580	672862	282	3
Clade4_00044	137948	138530	582	3
Clade4_00046	675757	676009	252	3
Clade4_00048	849764	849830	66	3
Clade4_00056	923188	923568	380	3
Clade4_00061	156431	157248	817	3
Clade4_00062	158525	159073	548	3

For clade 1 the start and end positions are based on the genomic positions of *L2b* strain.
For clade 2 the start and end positions are based on the genomic positions of *E/11023* strain.
For clade 3 the start and end positions are based on the genomic positions of *B/Tz* strain.
For clade 4 the start and end positions are based on the genomic positions of *D/UW3* strain.
